# Construction and verification of a risk prediction model for patients with kinesophobia after cerebral hemorrhage surgery

**DOI:** 10.1186/s12883-025-04296-0

**Published:** 2025-07-12

**Authors:** Yan Huang, Ya-ting Huang, Jie Yuan, Zhi-yu WuYang, Xue Zhang, Chuan-qing Yu

**Affiliations:** 1 Department of Neurosurgery, The First Affiliated Hospital of Anhui University of Science and Technology(First People’s Hospital of Huainan), Huainan, Anhui 232007 China; 2 Department of Interventional Catheterization Laboratory, The First Affiliated Hospital of Anhui University of Science and Technology(First People’s Hospital of Huainan), Huainan, Anhui 232007 China; 3 Department of Neurological Intensive Care Unit, The First Affiliated Hospital of Anhui University of Science and Technology(First People’s Hospital of Huainan), Huainan, Anhui 232007 China; 4 Department of Neurology, The First Affiliated Hospital of Anhui University of Science and Technology(First People’s Hospital of Huainan), Huainan, Anhui 232007 China

**Keywords:** Cerebral hemorrhage, Kinesophobia, Risk factors, Nomogram, Prediction model

## Abstract

**Objective:**

To establish a risk prediction model of kinesophobia in patients after cerebral hemorrhage surgery and verify its effect.

**Methods:**

A total of 218 patients after cerebral hemorrhage surgery were selected, and the differences in clinical data between kinesophobia patients and non-kinesophobia patients were analyzed. Using 20 indexes as independent variables, the characteristic variables were screened by LASSO regression, and then multivariate Logistic regression analysis was carried out. Based on the results, the nomogram prediction model was constructed, and the model was verified from the aspects of clinical applicability, discrimination, and calibration.

**Results:**

Significant differences were found in age, electronic health literacy score, depression score, NIHSS score, VAS pain score, intraoperative blood loss, and anxiety score between patients with phobia and non-phobia (*P* < 0.05). 12 characteristic variables were selected by LASSO regression. Multivariate Logistic regression analysis showed that age, NIHSS score, VAS pain score and depression score were independent risk factors for the occurrence of kinesophobia after cerebral hemorrhage surgery (OR > 1 and *P* < 0.05), and electronic health literacy score was an independent protective factor (OR < 1 and *P* < 0.05). Based on age, NIHSS score, VAS pain score, e-health literacy score, and depression score, a nomogram prediction model was constructed. The DCA curve shows that the model has the highest clinical net benefit when the threshold probability is between 0.14 and 0.99, indicating good clinical applicability. The area under the ROC curve (AUC) is 0.836(95% CI: 0.782–0.890), which indicates good discrimination. Spiegelhalter’s z test and the calibration curve show that the calibration degree is good, and the C statistic after Bootstrap self-sampling internal verification is 0.820 (95% CI: 0.772–0.877), indicating that the prediction is robust.

**Conclusion:**

The nomogram prediction model of the risk of kinesophobia after cerebral hemorrhage based on multivariate regression analysis has a good prediction effect, which can provide reference for the clinical prevention of kinesophobia after cerebral hemorrhage.

**Supplementary Information:**

The online version contains supplementary material available at 10.1186/s12883-025-04296-0.

## Introduction

Cerebral hemorrhage is a serious nervous system disease, which is usually caused by hypertension, cerebrovascular malformation, or trauma. Its clinical manifestations include headache, disturbance of consciousness, and local neurological deficit, and the disease develops rapidly, often leading to death or severe disability [1]. In terms of treatment, surgical intervention is a common choice, but the incidence of postoperative complications is high, among which kinesiophobia is an important psychological problem that may affect the rehabilitation of patients. Kinesiophobia is an excessive and irrational fear of physical movement or activity, often stemming from fear of pain, re-injury, or deteriorating health, leading to avoidance behaviors that can seriously affect recovery and quality of life [2]. Kinesiophobia has multifaceted impacts on patients who have undergone surgery for cerebral hemorrhage. Research indicates that postoperative avoidance of movement not only significantly delays the process of neurological recovery but also increases the risk of complications such as joint contractures and muscle atrophy [3]. Moreover, the overall incidence of postoperative complications after cerebral hemorrhage surgery is high, with kinesiophobia, as an important psychological complication, having an occurrence rate as high as 35.7% [4]. Additional studies show that the severity of postoperative consciousness impairment, bleeding volume, and location are directly related to mortality rates in cerebral hemorrhage patients [5], while kinesiophobia may indirectly exacerbate poor prognosis by suppressing compliance with rehabilitation.

The choice of cerebral hemorrhage patients as research subjects is primarily due to the specificity of their pathological characteristics and surgical requirements. Cerebral hemorrhage onset is abrupt, with a disability rate as high as 30-50% [5, 6], and H-type hypertension (hypertension combined with hyperhomocysteinemia) significantly elevates the risk of cerebral hemorrhage in patients [7, 8]. This population is more likely to develop kinesiophobia postoperatively due to interactions between physiological and psychological factors. The risk of kinesophobia after cerebral hemorrhage surgery is related to many factors, including postoperative physiological changes, psychological state, and social support. Studies have shown that early postoperative mental state assessment is very important for identifying high-risk patients [9]. In addition, inflammatory reaction and recovery of neurological function are also considered to be closely related to the development of kinesophobia, and these factors may increase the risk of kinesophobia by affecting the cognitive and emotional state of patients [10, 11]. 

Kinesophobia is an important psychological barrier that can significantly impact patients’ recovery process after cerebral hemorrhage. Current methods for evaluating kinesophobia primarily rely on self-reported questionnaires such as the Tampa Scale for Kinesiophobia (TSK), the Fear-Avoidance Beliefs Questionnaire (FABQ) and Kinesiophobia Cause Scale(KCS) [12–14]. While these tools provide valuable insights into patients’ subjective experiences, they have limitations in terms of objectivity and may not fully capture the complex interplay between psychological factors and physical functioning. Moreover, existing studies often focus on analyzing the current status and related factors of kinesophobia, failing to effectively integrate multiple factors for personalized prediction of kinesophobia risk.

The occurrence of kinesophobia in patients after cerebral hemorrhage surgery is a complex problem that requires a comprehensive consideration of physiological, psychological, and social support factors. Constructing an effective risk prediction model can help identify high-risk patients and provide a basis for formulating personalized treatment plans, thereby improving the prognosis of patients and promoting their rehabilitation. Therefore, in order to effectively predict the risk of kinesophobia in patients after cerebral hemorrhage, it is necessary to construct a prediction model that comprehensively considers various clinical indicators. This model can be evaluated based on the patient’s demographic characteristics, medical history, and surgical conditions. For example, studies have shown that factors such as old age, high blood pressure, and previous stroke history can significantly increase the risk of recurrent cerebral hemorrhage, and these factors may also affect mental health, thus indirectly leading to kinesophobia [15, 16]. Therefore, this study aims to provide valuable information for clinicians to take early intervention measures by establishing a prediction model containing these key variables. The results are reported as follows.

## Objects and methods

### Research objects

A cross-sectional study was conducted to screen patients with cerebral hemorrhage admitted to the Department of Neurosurgery of the First Affiliated Hospital of Anhui University of Science and Technology from November to December 2024. Information was collected from qualified subjects after they signed the informed consent form. Inclusion criteria: (1) Meet the diagnostic criteria for cerebrovascular disease [17]. The diagnosis of cerebral hemorrhage was confirmed by CT/MRI. (2) Patients had indications for surgery and had undergone the procedure; (3) Patients were conscious, stable, and able to cooperate in completing the questionnaire survey; (4) Patients were aged > 18 years; (5) Patients had adequate reading and expressive abilities; (6) Patients voluntarily participated in the study and signed the informed consent form. Exclusion criteria: (1) Patients with venous-related diseases such as varicose veins, phlebitis, and chronic venous insufficiency; (2) Patients with coagulation disorders who have abnormal bleeding in other parts of the body. The criteria for after cerebral hemorrhage surgery was clarified as follows: it refers to the one week post-surgery period. A stable state is considered when vital signs such as blood pressure, respiration, and body temperature are stable; GCS score is ≥ 13 with no progressive loss of consciousness. This study protocol has been approved by the Ethics Committee of the First Affiliated Hospital of Anhui University of Science and Technology, ensuring compliance with the principles outlined in the Declaration of Helsinki. All participants signed an informed consent form prior to their involvement in the study, safeguarding their right to be informed and their voluntary participation.

### Research tools

1. General information questionnaire. A self-made questionnaire was used, referring to the relevant indicators in related study [4], which mainly included the demographic data, complications, and operation-related data of patients. Specifically, it covered gender, age, education level, marital status, type of residence, payment method of medical expenses, bleeding location, type of operation, duration of operation, intraoperative blood loss, postoperative hypokalemia, coronary heart disease, respiratory diseases(including chronic obstructive pulmonary disease, chronic pneumonia, asthma), hypertension, and diabetes. The self-made general information questionnaire is provided in Supplementary File 1.
2. Hospital Anxiety and Depression Scale (HADS) [18]. It contains 14 items, with 7 items for anxiety and 7 items for depression. The questionnaire uses a Likert 4-point scale, with scores ranging from 0 to 7 indicating no anxiety/depression, 8 to 10 indicating possible anxiety/depression symptoms, and ≥ 11 indicating significant mental health issues. The Chinese version of this scale has a Cronbach’s α coefficient of 0.866 and a Kappa value of 0.314, demonstrating good reliability and validity [19]. It is effective for screening depressive and anxiety symptoms in hospitalized patients [20].
3. eHealth Literacy Scale (eHEALS): This scale was developed by Norman [21]. It was adapted and introduced in Chinese by Guo Shuaijun et al. [22] The scale assesses the ability of individuals to obtain health information through the Internet and use it to address health problems. It includes three dimensions: application ability of online health information and services (5 items), judgment ability (2 items), and decision-making ability (1 item), with a total of 8 items. Each item uses a Likert 5-point scale, ranging from 1 (“very inconsistent”) to 5 (“very consistent”), with a total score of 8 to 40 points. Higher scores indicate higher levels of eHealth literacy. The Cronbach’s α coefficient of the scale is 0.91 [23].
4. Tampa Scale for Kinesiophobia (TSK-11): The Chinese version of TSK-11 was translated and validated by Cai Libai et al. [24] The scale has a Cronbach’s α coefficient of 0.883 and a test-retest reliability of 0.798 [25]. It consists of 11 items in a single dimension. The scoring system uses a Likert 4-point scale, with 1 point for “Strongly Disagree,” 2 points for “Disagree,” 3 points for “Agree,” and 4 points for “Strongly Agree,” with a total score ranging from 11 to 44. A score above 26 indicates the presence of kinesiophobia. [24] Based on the TSK-11 scores, the research subjects were divided into two groups: 103 patients with kinesophobia (score > 26) and 115 patients without kinesophobia (score ≤ 26).
5. National Institutes of Health Stroke Scale (NIHSS) score: This scale consists of 11 items, including level of consciousness, gaze, visual fields, facial palsy, upper limb motor function, lower limb motor function, ataxia, sensation, language, dysarthria, and neglect, with a total score ranging from 0 to 42. A higher score indicates poorer neurological function [26]. The Cronbach’s α coefficient for this scale is 0.921 [27]. The NIHSS score analyzed in this study is the score obtained 24 h postoperatively.
6. Postoperative pain in patients with cerebral hemorrhage: Postoperative pain in patients with cerebral hemorrhage was assessed using the Visual Analog Scale (VAS). The assessment method involves selecting a ruler marked with uniform gradations (0 to 10), with the scale facing away from the patient. The patient then selects a point on the ruler that best represents their level of pain, and the healthcare provider reads the corresponding value, which is the VAS pain score. A score of 0 indicates no pain; scores below 3 indicate mild pain that is tolerable and does not affect sleep or daily life; scores between 4 and 6 indicate moderate pain that is still tolerable but affects sleep; scores from 7 to 10 indicate severe pain that is difficult to tolerate and affects appetite and sleep [28]. The final pain score is the average of the highest and lowest pain scores recorded over the past two weeks.

### Data collection

The survey results consist of electronic medical records and questionnaires. The researcher distributed relevant questionnaires one day before the patient was discharged from the hospital. Initially, the researcher explained the purpose and precautions of the study to the patient and asked the patient to fill it out truthfully. If there were any doubts, the researcher answered them on the spot. If the patient was unable to fill it out by themselves, the researcher would objectively describe the questions to the patient, who would then provide truthful answers. The researcher would then record the responses on the questionnaire according to the patient’s answers. After the questionnaire was completed, the researcher checked its completeness and validity on the spot and addressed any omissions. In this study, 220 questionnaires were distributed, and 218 valid questionnaires were recovered, resulting in an effective recovery rate of 99.09%.

### Statistical methods

Statistical software Stata 17.0 and R software (R 3.6.1) were used for statistical analysis. Counting data were expressed as rates. For comparisons between two groups, the chi-square test was employed. Measurement data that followed a normal distribution were expressed as (mean ± standard deviation), and comparisons between the two groups were made using the t-test. Measurement data with a skewed distribution were expressed as M(P25, P75), and the Mann-Whitney U test was used for comparisons between the two groups. To identify the characteristic variables associated with kinesophobia, LASSO regression was applied. This method is particularly advantageous in scenarios where there are multiple potential predictors, as it not only helps in reducing model complexity but also performs variable selection by shrinking some coefficients to zero. The LASSO regression was implemented using cross-validation to determine the optimal value of the regularization parameter Lambda, which controls the degree of shrinkage applied to the regression coefficients. The model with the smallest cross-validation error was identified as the optimal model, indicating the best balance between bias and variance. The independent risk factors for kinesophobia were determined using a multivariate logistic regression model. The “nomologit” package in R software (R 3.6.1) was used to create nomograms. The area under the receiver operating characteristic (ROC) curve is used to evaluate the discrimination of the nomogram. The Spiegelhalter’s z test and the calibration curve were used to evaluate the calibration of the nomogram. The decision curve analysis(DCA) curve is used to evaluate the clinical applicability of the nomogram model. To prevent overfitting of the nomogram model, Bootstrap self-sampling was performed 500 times for internal validation. To further enhance the interpretability of the nomogram model, we conducted SHapley Additive exPlanations (SHAP) analysis on the nomogram model. The purpose of SHAP analysis is to explain the prediction results of predictive models by quantifying the contribution of each feature to individual predictions, thereby helping to understand the decision-making basis of the model and enhancing its interpretability. A *p*-value of less than 0.05 was considered statistically significant.

## Results

### Comparison of clinical data between patients with kinesophobia and patients without kinesophobia

The average age of the 218 subjects was 59.99 ± 10.58 years, including 106 males and 112 females. See Table 1 for other clinical data. The prevalence rate of kinesophobia was 47.25%. When comparing the clinical data between patients with kinesophobia and those without, statistically significant differences were observed in seven indicators, including age, electronic health literacy score, depression score, intraoperative blood loss, NIHSS score, VAS pain score and anxiety score (*P* < 0.05). These results are detailed in Table 1.

**Table 1:** Comparison of clinical data between patients with kinesophobia and patients without kinesophobia

Variables	Total (*n* = 218)	Non-kinesophobia group (*n* = 115)	Kinesophobia group (*n* = 103)	Statistic	*P*
Age, mean ± SD	59.99 ± 10.58	57.03 ± 10.77	63.28 ± 9.36	t=−4.55	**< 0.001**
Total score of electronic health literacy, mean ± SD	24.40 ± 5.91	26.07 ± 5.16	22.54 ± 6.15	t = 4.60	**< 0.001**
Depression score, mean ± SD	6.45 ± 2.63	5.41 ± 1.77	7.62 ± 2.93	t=−6.64	**< 0.001**
Operation duration (minutes), M (Q_1_, Q_3_)	120.00 (120.00, 180.00)	120.00 (120.00, 180.00)	120.00 (110.00, 180.00)	Z=−0.37	0.71
Intraoperative blood loss, M (Q_1_, Q_3_)	25.00 (20.00, 50.00)	20.00 (20.00, 50.00)	30.00 (20.00, 80.00)	Z=−2.04	**0.041**
Anxiety score, M (Q_1_, Q_3_)	7.00 (6.00, 8.00)	7.00 (5.00, 7.00)	8.00 (7.00, 10.00)	Z=−6.05	**< 0.001**
NIHSS score, M (Q_1_, Q_3_)	9.00 (4.00, 18.00)	8.00 (4.00, 13.50)	10.00 (4.00, 22.00)	Z=−2.15	**0.031**
VAS pain score, M (Q_1_, Q_3_)	4.00 (2.00, 6.00)	3.00 (2.00, 5.00)	5.00 (3.00, 8.00)	Z=−4.00	**< 0.001**
Hypokalemia, n(%)				χ^2^=0.30	0.581
No	212 (97.25)	113 (98.26)	99 (96.12)		
Yes	6 (2.75)	2 (1.74)	4 (3.88)		
Gender, n(%)				χ^2^=0.27	0.603
Male	106 (48.62)	54 (46.96)	52 (50.49)		
Female	112 (51.38)	61 (53.04)	51 (49.51)		
Education level, n(%)				χ^2^=2.14	0.143
Junior high school and below	122 (55.96)	59 (51.30)	63 (61.17)		
High school, technical secondary school and above	96 (44.04)	56 (48.70)	40 (38.83)		
Marital status, n(%)				χ^2^=0.84	0.359
No spouse	7 (3.21)	2 (1.74)	5 (4.85)		
Have a spouse	211 (96.79)	113 (98.26)	98 (95.15)		
Type of operation, n(%)				χ^2^=9.37	0.052
Endovascular coiling for aneurysm or craniotomy for aneurysm clipping	102 (46.79)	61 (53.04)	41 (39.81)		
Craniotomy for hematoma evacuation	64 (29.36)	35 (30.43)	29 (28.16)		
Stereotactic-guided intracerebral hematoma puncture and drainage	20 (9.17)	6 (5.22)	14 (13.59)		
Ventricular drainage and clot lysis	21 (9.63)	10 (8.70)	11 (10.68)		
Decompressive craniectomy	11 (5.05)	3 (2.61)	8 (7.77)		
Residence, n(%)				χ^2^=3.19	0.074
Village	90 (41.28)	41 (35.65)	49 (47.57)		
Cities and towns	128 (58.72)	74 (64.35)	54 (52.43)		
Payment method of medical expenses, n(%)				χ²=0.24	0.623
Pay one’s own expenses	13 (5.96)	6 (5.22)	7 (6.80)		
Medical insurance reimbursement	205 (94.04)	109 (94.78)	96 (93.20)		
Complicated with coronary heart disease, n(%)				χ^2^=2.52	0.112
No	173 (79.36)	96 (83.48)	77 (74.76)		
Yes	45 (20.64)	19 (16.52)	26 (25.24)		
Complicated with respiratory diseases, n(%)				χ^2^=1.62	0.204
No	181 (83.03)	99 (86.09)	82 (79.61)		
Yes	37 (16.97)	16 (13.91)	21 (20.39)		
Complicated with hypertension, n(%)				χ^2^=0.03	0.861
No	96 (44.04)	50 (43.48)	46 (44.66)		
Yes	122 (55.96)	65 (56.52)	57 (55.34)		
Combined with diabetes, n(%)				χ^2^=1.01	0.316
No	145 (66.51)	73 (63.48)	72 (69.90)		
Yes	73 (33.49)	42 (36.52)	31 (30.10)		
Bleeding site, n(%)				χ^2^=2.59	0.763
Basal nuclei	43 (19.72)	20 (17.39)	23 (22.33)		
Brainstem	13 (5.96)	9 (7.83)	4 (3.88)		
Ventricle of the brain	89 (40.83)	46 (40.00)	43 (41.75)		
Brain lobe	38 (17.43)	22 (19.13)	16 (15.53)		
Thalamencephalon	18 (8.26)	9 (7.83)	9 (8.74)		
Cerebellum	17 (7.80)	9 (7.83)	8 (7.77)		

*t* t-test, *Z* Mann-Whitney test, *χ*^2^ Chi-square test, *SD* standard deviation, *M* Median, *Q*_1_ 1 st Quartile, *Q*_3_ 3 st Quartile, Boldface values indicate statistical significance (*P* < 0.05)

### Screening of characteristic variables of kinesophobia in patients after cerebral hemorrhage surgery

We began by considering 20 indicators listed in Table 1 as independent variables for LASSO regression. In this case, the Lambda value corresponding to the minimum cross-validation error was found to be 0.023. At this specific Lambda value, 12 out of the initial 20 indicators were selected as significant characteristic variables due to their non-zero regression coefficients. These 12 variables included gender, age, type of surgery, presence of coronary heart disease, respiratory diseases, diabetes, intraoperative blood loss, electronic health literacy score, anxiety score, NIHSS score, VAS pain score and depression score. Each of these variables plays a distinct role in contributing to the risk or protective effect against kinesophobia after cerebral hemorrhage surgery.

### The results of multivariate logistic regression analysis on the occurrence of kinesophobia in patients after cerebral hemorrhage surgery

The results from the logistic regression analysis provided insights into how each of these variables based on the LASSO regression independently contributes to the likelihood of developing kinesophobia after cerebral hemorrhage surgery. Specifically, age, depression score, NIHSS score and VAS pain score emerged as significant independent risk factors for kinesophobia (with odds ratios greater than 1 and *p*-values less than 0.05). This suggests that older individuals, those with higher levels of pre-existing depressive symptoms, higher NIHSS scores, and elevated VAS pain scores are at increased risk of experiencing kinesophobia post-surgery. Conversely, the electronic health literacy score was identified as an independent protective factor against kinesophobia (with an odds ratio less than 1 and a *p*-value below 0.05). This finding implies that individuals who possess higher levels of electronic health literacy may have lower chances of developing kinesophobia. Detailed results are presented in Table 2.

**Table 2:** The results of multivariate logistic regression analysis on the occurrence of kinesophobia in patients after cerebral hemorrhage surgery

Variables	OR (95%CI)	*P*
Intercept	0.02 (0.00 ~ 0.71)	0.033
Age	1.04 (1.01 ~ 1.09)	**0.038**
Intraoperative bleeding volume	1.00 (1.00 ~ 1.01)	0.394
Total score of e-health literacy	0.89 (0.82 ~ 0.96)	**0.004**
Anxiety score	1.05 (0.84 ~ 1.32)	0.655
Depression score	1.49 (1.13 ~ 1.98)	**0.005**
NIHSS score	1.06 (1.02 ~ 1.10)	**0.004**
VAS pain score	1.19 (1.05 ~ 1.35)	**0.008**
Type of operation		
Endovascular coiling for aneurysm or craniotomy for aneurysm clipping	1.00 (Reference)	
Craniotomy for hematoma evacuation	1.03 (0.45 ~ 2.36)	0.952
Stereotactic-guided intracerebral hematoma puncture and drainage	1.45 (0.37 ~ 5.70)	0.595
Ventricular drainage and clot lysis	0.93 (0.28 ~ 3.02)	0.898
Decompressive craniectomy	4.18 (0.82 ~ 21.37)	0.086
Gender		
Male	1.00 (Reference)	
Female	0.60 (0.30 ~ 1.22)	0.156
Complicated with coronary heart disease		
No	1.00 (Reference)	
Yes	2.07 (0.76 ~ 5.67)	0.155
Complicated with respiratory diseases		
No	1.00 (Reference)	
Yes	2.71 (0.87 ~ 8.45)	0.087
Complicated with diabetes		
No	1.00 (Reference)	
Yes	0.67 (0.30 ~ 1.47)	0.316

*OR* Odds Ratio, *CI* Confidence Interval, Boldface values indicate statistical significance (*P* < 0.05)

### Construction of a nomogram prediction model for the risk of kinesophobia in patients after cerebral hemorrhage surgery

**Fig. 1:**
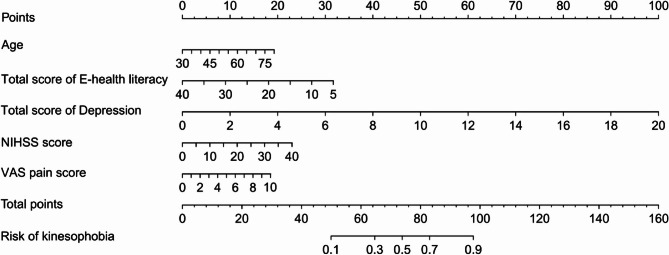
The nomogram prediction model of the risk of kinesophobia in patients after cerebral hemorrhage surgery

According to the results of the multivariate Logistic regression analysis in Table 2, using age, electronic health literacy score, NIHSS score, VAS pain score and depression score as predictive indicators, the “nomologit” package in R software (R 3.6.1) was used to construct a nomogram prediction model for the risk of kinesophobia after cerebral hemorrhage, as shown in Fig. 1. The nomogram can be interpreted as follows: (1) for each variable, draw a straight line up to the points axis to determine the points for that variable, (2) repeat this process for each variable, (3) add the points for all variables and locate the sum on the total points axis, and (4) draw a straight line from total points down to risk. To enhance the interpretability of the nomogram model, we developed an interpretable risk scoring system based on the nomogram model, detailed in Table 3.

**Table 3:** The interpretable risk scoring system of the nomogram

Variables	Points
Age	
30	0
35	2
40	4
45	6
50	8
55	10
60	12
65	13
70	15
75	17
80	19
Total score of E-health literacy	
5	32
10	27
15	23
20	18
25	14
30	9
35	5
40	0
Total score of Depression	
0	0
2	10
4	20
6	30
8	40
10	50
12	60
14	70
16	80
18	90
20	100
NIHSS Score	
0	0
5	3
10	6
15	9
20	12
25	14
30	17
35	20
40	23
VAS Pain Score	
0	0
1	2
2	4
3	6
4	7
5	9
6	11
7	13
8	15
9	17
10	19

Total Points	Risk of kinesophobia
<50	<0.1
50	0.1
59	0.2
65	0.3
69	0.4
74	0.5
78	0.6
83	0.7
89	0.8
98	0.9
>98	>0.9

### Evaluation of clinical applicability of the nomogram model for the risk of kinesophobia

**Fig. 2:**
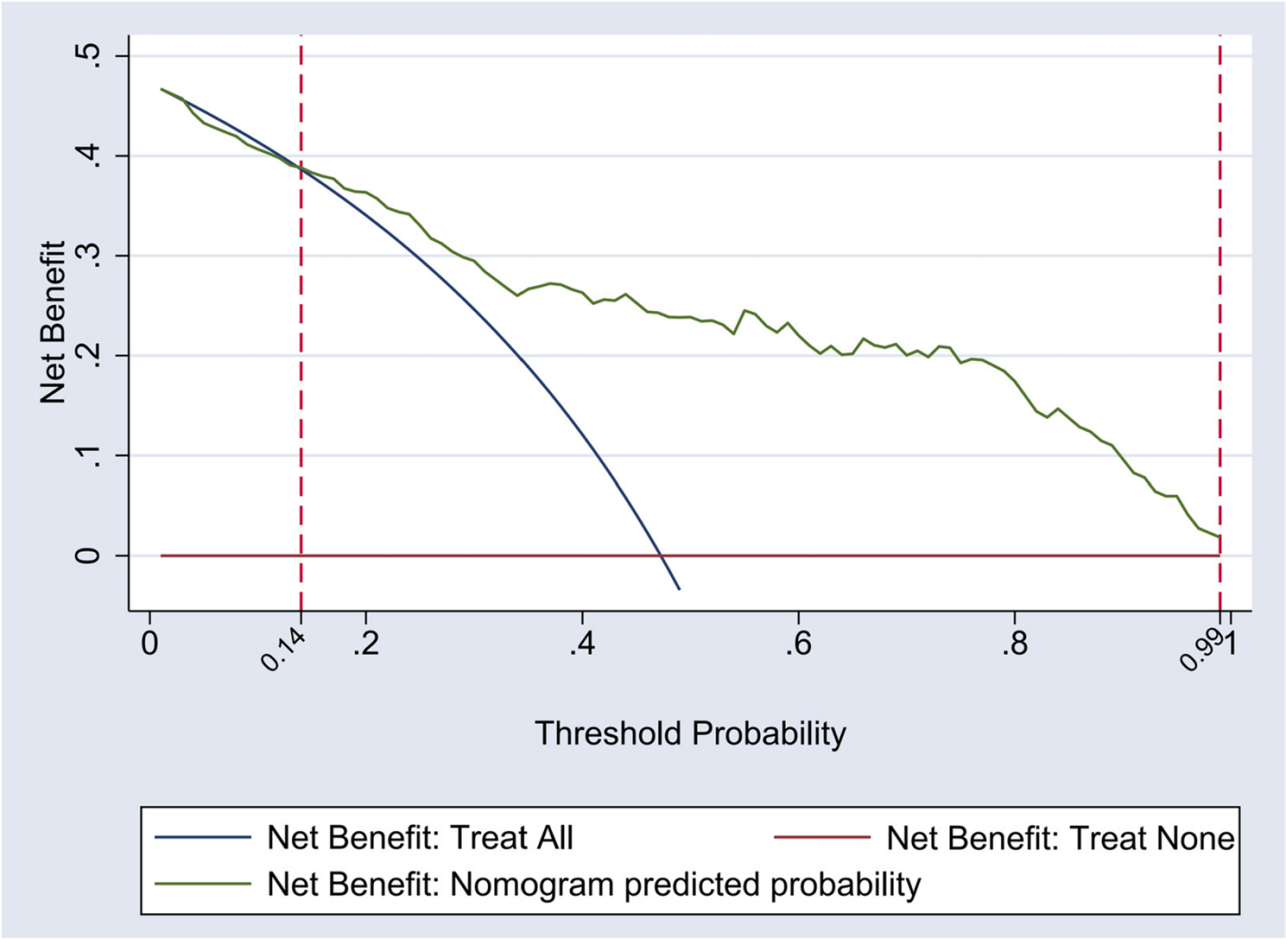
DCA curve of the nomogram model

The results of DCA curve of the nomogram show that when the threshold probability of kinesophobia in patients after cerebral hemorrhage is between 0.14 and 0.99, the application of the nomogram model can provide the highest clinical net benefit level for patients, which is superior to the “full intervention” and “no intervention” strategies. This suggests that the nomogram model has good clinical applicability. See Fig. 2 for details.

### Evaluation of discrimination and calibration of the nomogram model

**Fig. 3:**
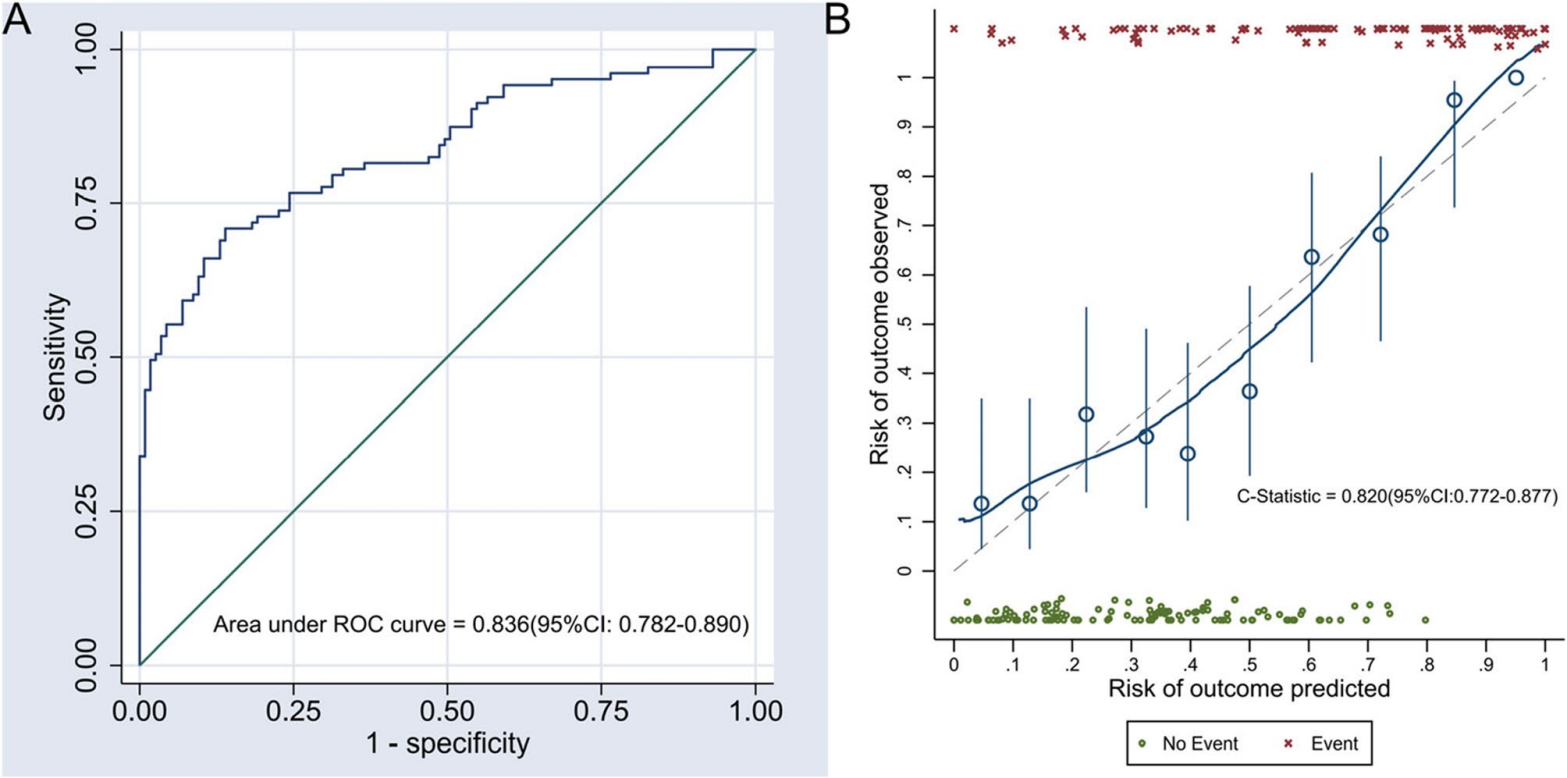
ROC curve and calibration curve of the nomogram model. Note: **A** ROC curve of the nomogram model before internal validation; **B** Calibration curve of the nomogram model after internal validation

The result shows that the AUC of the nomogram model is 0.836 (95% CI: 0.782–0.890), indicating that it has good discrimination. See Fig. 3A for details.

The results showed that the deviation between the prediction probability of the nomogram model and the actual frequency of kinesophobia was not statistically significant (z = −0.260, *P*-value = 0.603), indicating that the nomogram had good calibration.

The C statistic of the nomogram model after internal validation was 0.820 (95% CI: 0.772–0.877), and the calibration curve after internal validation also indicated that the nomogram had good calibration, suggesting that the nomogram has good prediction robustness. See Fig. 3B for details.

### SHAP analysis of the nomogram model

**Fig. 4:**
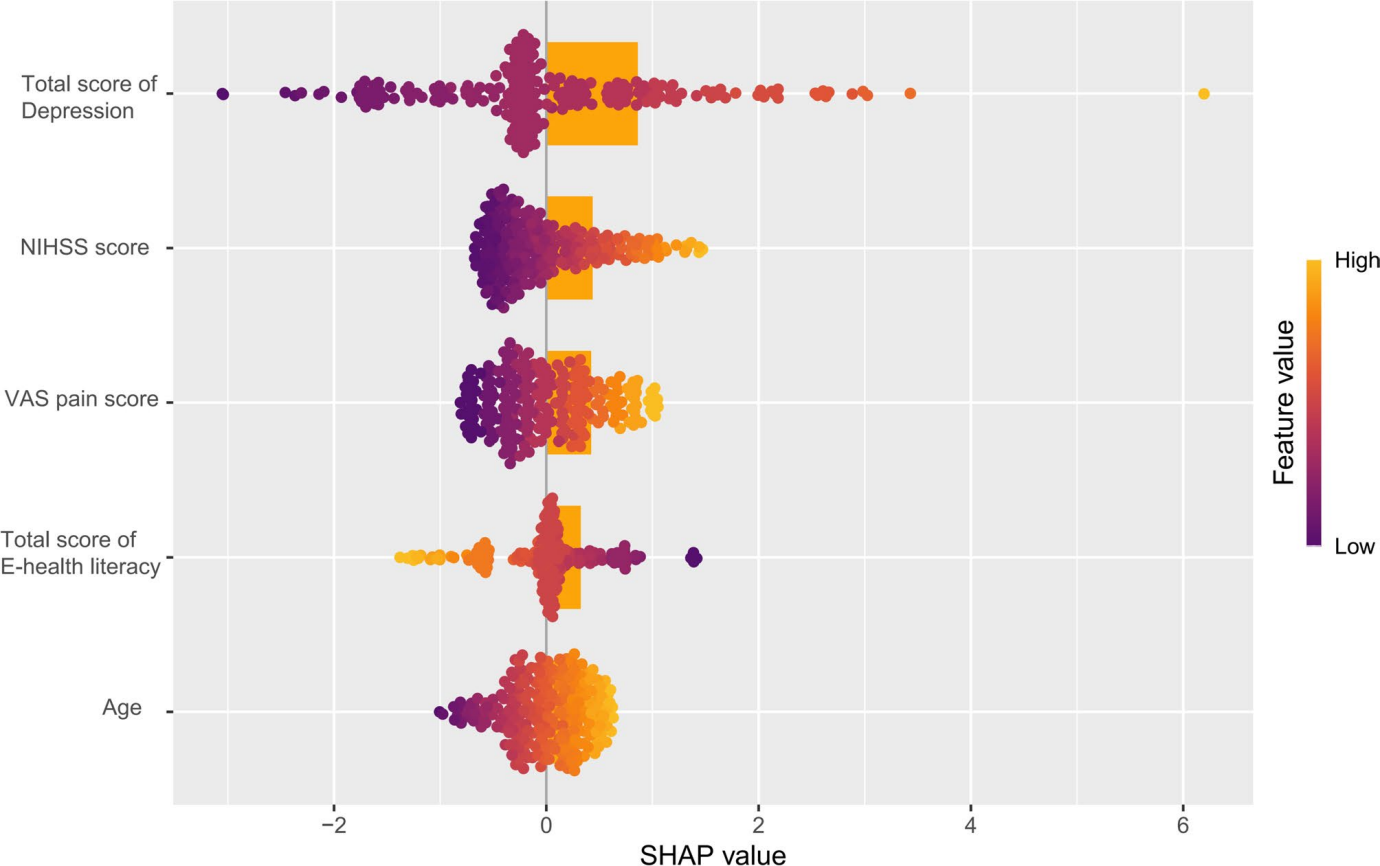
SHAP importance plot of the nomogram model

In Fig. 4, the SHAP importance plot of the nomogram model shows a color transition from purple (low feature values) to yellow (high feature values), which further illustrates the relationship between feature values and SHAP values, aiding our understanding of how each feature influences model predictions. That is, Fig. 4 demonstrates the impact of five indicators on the prediction of kinesophobia by the nomogram model and their order of importance. Based on the distribution of SHAP values (x-axis) and feature values (color) in Fig. 4, the following conclusions can be drawn: the indicators ranked from most to least important are Total score of Depression, NIHSS score, VAS pain score, Total score of E-health literacy, and Age. Among these, Total score of Depression, NIHSS score, VAS pain score, and Age have positive effects on kinesophobia, meaning that higher values of these indicators result in stronger positive impacts on predicting kinesophobia. On the other hand, Total score of E-health literacy has a negative effect on kinesophobia; that is, higher values of Total score of E-health literacy result in lower SHAP values, leading to stronger negative impacts on predicting kinesophobia.

**Fig. 5:**
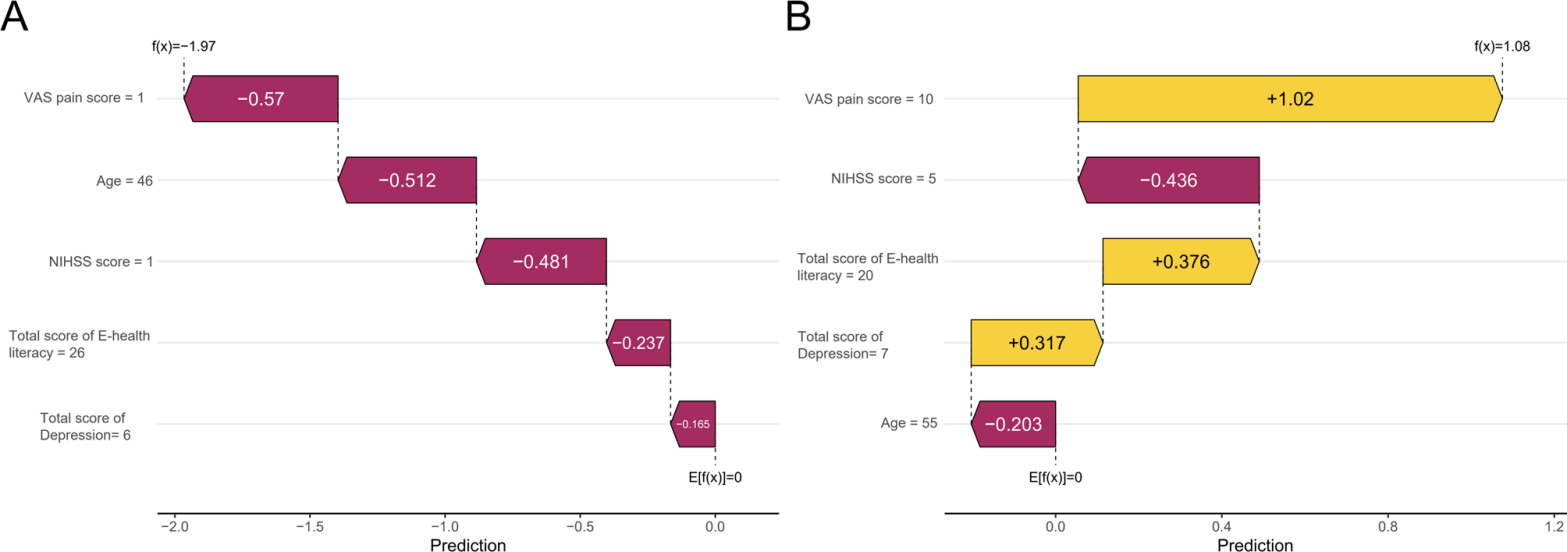
Analysis of SHAP waterfall plots for the nomogram model. Note: **A** Individual efforts by patients without kinesophobia; **B** Individual efforts by patients with kinesophobia

The SHAP waterfall plots (Fig. 5) analyzes how features influence the target variable by showing the contribution of each feature to the model’s prediction for an individual. In the SHAP waterfall plot for individuals without kinesophobia (Fig. 5A), ‘VAS pain score = 1’ decreases the SHAP predicted value by 0.57, ‘Age = 46’ decreases it by 0.512, ‘NIHSS score = 1’ decreases it by 0.481, ‘Total score of E-health literacy = 26’ decreases it by 0.237, and ‘Total score of Depression = 6’ decreases it by 0.165. These five indicators, at their current values, all have a negative impact on the occurrence of kinesophobia, causing the SHAP predicted value to decrease from the baseline of 0 to −1.97, indicating that the combined effect of these indicators reduces the likelihood of kinesophobia. Individuals without kinesophobia had a low SHAP prediction score (−1.97). In the SHAP waterfall plot for individuals with kinesophobia (Fig. 5B), ‘VAS pain score = 10’ increases the SHAP predicted value by 1.02; ‘NIHSS score = 5’ decreases it by 0.436; ‘Total score of E-health literacy = 20’ increases it by 0.376; ‘Total score of Depression = 7’ increases it by 0.317; and ‘Age = 55’ decreases it by 0.203. Overall, the positive effects of three indicators and the negative effects of two indicators interact, resulting in the SHAP predicted value increasing from the baseline of 0 to 1.08, indicating that the combined effect of these indicators increases the likelihood of kinesophobia. Individuals with kinesophobia had a high SHAP prediction score (1.08).

## Discussion

### Analysis of the prevalence of kinesophobia in patients after cerebral hemorrhage surgery

In this study, the prevalence rate of kinesophobia in 218 patients after cerebral hemorrhage surgery was 47.25%, which was higher than that reported in previous studies (35.7%) [4]. Kinesophobia is widely prevalent among surgical patients, consistent with the incidence of kinesophobia (21.8–58.4%) after total knee replacement [29, 30]. However, it is higher than the incidence in patients with breast cancer after surgery (29.59%) [31]. It is also lower than the incidence in patients with lumbar disc herniation after surgery (78.62%) [32]. Additionally, it is lower than the incidence in school-age children after fracture surgery (96.92%) [33]. The variation in the incidence of kinesophobia among surgical patients may be related to differences in disease type, sample size, and pain nature. This highlights the need for clinical medical staff to pay close attention to kinesophobia and implement appropriate interventions based on the specific disease. For patients with cerebral hemorrhage, early rehabilitation is crucial for promoting neurological recovery and preventing complications [34]. The presence of kinesophobia can negatively impact the timing and content of early rehabilitation exercises, which is detrimental to functional recovery [35]. Given the limited research on kinesophobia in patients with cerebral hemorrhage, further attention and investigation are warranted.

### Analysis of independent influencing factors of kinesophobia in patients after cerebral hemorrhage surgery

The results of the multivariate Logistic regression analysis in this study showed that age was an independent risk factor for the occurrence of kinesophobia in postoperative patients with cerebral hemorrhage (OR = 1.04, *P* = 0.038). This finding is consistent with the research by Lv Fulong et al., which also confirmed that age is one of the factors contributing to kinesiophobia in postoperative patients (*P* < 0.05) [36]. Older patients may have poorer physical function and weaker recovery abilities, and are more likely to worry about uncertainties during the surgical and rehabilitation processes, making them more susceptible to panic attacks [37]. In addition, with the increase of age, the central nervous system, muscle strength and balance ability of human body gradually degenerate. This physiological recession directly leads to the weakening of exercise ability, increases the risk of falling, and thus triggers excessive concern about exercise [38]. The cognitive bias of the elderly towards the risks of exercise also plays a key role, such as excessive concern about exercise-induced cardiovascular and cerebrovascular events [39]. This psychology forms a vicious cycle with physical decline, ultimately making age the core driving factor for kinesiophobia. The electronic health literacy score was identified as an independent protective factor against kinesophobia after cerebral hemorrhage surgery (OR = 0.89, *P* = 0.004). This result aligns with the findings of Lv Wenjing et al.‘s study on 116 acute myocardial infarction patients, which showed a negative correlation between total e-health literacy scores and total kinesiophobia scores (*r* = −0.629, *P* < 0.001) [40]. This indicates that higher levels of e-health literacy are associated with lower degrees of kinesiophobia. Patients with higher e-health literacy can better obtain and understand disease-related information and have a more accurate understanding of postoperative rehabilitation, thereby reducing the incidence of kinesophobia [40]. In addition, e-health literacy is positively correlated with self-management ability. Patients with high level of e-health literacy can better formulate and implement health plans, thus enhancing their sense of control over diseases or health risks and reducing kinesiophobia caused by uncertainty [41]. The depression score was an independent risk factor for kinesophobia in patients after cerebral hemorrhage surgery (OR = 1.49, *P* = 0.005). This finding is supported by the research of Zhang Xiuyan et al., which revealed a positive correlation between kinesiophobia scores and depression in postoperative patients [42]. Depression can affect patients’ psychological state and coping abilities, making them more fearful of pain and the rehabilitation process, thus increasing the risk of kinesophobia [43]. Patients with depression often accompany negative coping styles, such as avoiding problems or self-attack tendencies, which significantly reduce individuals’ motivation to participate in activities [44, 45]. Evidence shows that negative coping styles are positively correlated with depressive symptoms, aggressive behavior and anxiety levels, while positive coping styles are negatively correlated with them [41, 42]. When patients have negative expectations for the results of an activity, they may avoid exercise due to fear of failure or increased pain, forming a cognitive basis for kinesiophobia. Other studies have shown that depressive mood can lead to cognitive impairment, such as decreased attention and reduced executive function [46], which weakens patients’ ability to plan and execute movements, increasing frustration. This study found that the VAS pain score is an independent risk factor for kinesiophobia in patients after cerebral hemorrhage surgery (OR = 1.19, *P* = 0.008), indicating a significant positive correlation between subjective pain perception and the risk of kinesiophobia. This finding aligns with the conclusions of Fagevik et al., [47] confirming the significant impact of postoperative pain intensity on the occurrence of kinesiophobia. Patients after cerebral hemorrhage surgery often experience varying degrees of pain symptoms, which may be related to surgical trauma and hemodynamic changes in the brain caused by the disease itself. Fluctuations in intracranial pressure may be the primary cause of pain, while factors such as changes in body position, activity, and emotional fluctuations can induce changes in intracranial pressure, leading to recurrent pain. This pain experience can easily lead to conditioned reflexes, causing patients to develop a fear of activities that might trigger pain even after the pain has subsided [48], thereby increasing the risk of kinesiophobia and significantly affecting postoperative rehabilitation and treatment outcomes. It is particularly noteworthy that headache, a common symptom in cerebral hemorrhage patients, may persist postoperatively. This persistent pain stimulus can further exacerbate the patient’s movement fear, creating a vicious cycle. Therefore, in clinical practice, postoperative pain management should be emphasized, effectively controlling pain to reduce the risk of kinesiophobia and promote patient recovery. In this study, the NIHSS score was identified as an independent risk factor for kinesiophobia in patients after cerebral hemorrhage surgery (OR = 1.06, *P* = 0.004), indicating that a higher NIHSS score correlates with an increased risk of postoperative kinesiophobia. The NIHSS score is a crucial tool for assessing the degree of neurological deficits in stroke patients, with higher scores indicating more severe neural damage [49]. For patients after cerebral hemorrhage surgery, a high NIHSS score indicates significant neurological deficits (such as limb weakness, balance disorders, or sensory abnormalities), leading to a marked decrease in mobility [50], which can result in feelings of frustration and fear during rehabilitation training due to movement difficulties. Additionally, neurological damage can affect cognitive and emotional regulation abilities [51], increasing sensitivity to pain and movement risks, thereby reinforcing avoidance behaviors. Furthermore, patients with severe neurological deficits often lack confidence in their rehabilitation prognosis, fearing that activity may exacerbate their condition or lead to complications, and they experience higher levels of disease-related stigma [52], which also significantly increases the risk of kinesiophobia. Therefore, in clinical practice, for patients with high NIHSS scores after cerebral hemorrhage surgery, psychological intervention and gradual rehabilitation training should be strengthened to reduce the incidence of kinesiophobia and promote functional recovery.

In addition, there may be some relationship between different surgical types and the occurrence of kinesophobia. Although some surgical types did not show significant differences in the multivariate Logistic regression analysis, the OR value for decompressive craniectomy was relatively high (OR = 4.18, *P* = 0.086), which may be related to the greater trauma and more pronounced impact on patients. Moreover, the presence of comorbidities such as coronary heart disease and respiratory diseases may increase the risk of kinesophobia in patients after cerebral hemorrhage surgery, although the OR values were not statistically significant in the multivariate Logistic regression analysis. For example, the OR values for patients with coronary heart disease (OR = 2.07, *P* = 0.155) and respiratory diseases (OR = 2.71, *P* = 0.087) were both greater than 1, suggesting that these combined diseases may increase the physical burden on patients, affect the rehabilitation process, and lead to panic attacks.

In this study, intraoperative blood loss was not found to be statistically significant in relation to the occurrence of kinesophobia (OR = 1.00, *P* = 0.394); however, substantial blood loss may potentially increasing the likelihood of kinesophobia. Specifically, Cui et al.‘s study on patients with thoracolumbar fractures found that excessive blood loss during surgery and longer postoperative recovery time led to reduced muscle strength in patients, making it more difficult for them to engage in rehabilitation exercises and increasing the likelihood of developing kinesiophobia [53]. 

### Significance analysis of the risk prediction model for patients with kinesophobia after cerebral hemorrhage surgery

The nomogram is a tool that predicts the probability of an event through an intuitive graphic display based on multiple predictive variables. It is intuitive, easy to understand, and easy to calculate, and can integrate multi-factor information. It can help medical staff quickly assess the risk level of patients and provide a basis for formulating personalized intervention measures [54]. Previous studies have shown that nomograms can effectively predict stroke-related pneumonia in patients with cerebral hemorrhage [55]. They have also been used to predict the risk of cerebral hemorrhage in patients with carotid stenosis undergoing stent implantation [56]. However, no studies have reported on the nomogram prediction model for the risk of kinesophobia in patients after cerebral hemorrhage surgery. Constructing a nomogram prediction model for this purpose is of great significance.

From a clinical perspective, it provides an intuitive and convenient tool for medical staff to help them quantitatively evaluate the risk of kinesophobia in patients in a short time, thereby enabling the formulation of personalized intervention measures and achieving precise medical care. For patients, accurate risk prediction helps them understand their own condition, prepare psychologically, and actively participate in treatment and rehabilitation training. From the perspective of medical resource allocation, screening high-risk patients through prediction can focus medical resources more efficiently on those who truly need them, improving resource utilization and avoiding unnecessary waste.

The nomogram prediction model constructed in this study demonstrates good predictive performance. The DCA curve was used to evaluate its clinical applicability. The results show that when the threshold probability of panic attacks in patients after cerebral hemorrhage is between 0.14 and 0.99, the application of this model can provide the highest clinical net benefit level for patients, which is superior to both the “full intervention” and “no intervention” strategies. This fully demonstrates the practical value of the model in clinical practice.

In terms of discrimination, the area under the ROC curve is 0.836(95% CI: 0.782–0.890), indicating that the model can effectively distinguish between patients with and without panic attacks. Regarding calibration, Spiegelhalter’s z test results show that the deviation between the predicted probability of the nomogram model and the actual frequency of kinesophobia is not statistically significant (z = −0.260, *P*-value = 0.603). The calibration curve after 500 internal validations using Bootstrap self-sampling also indicates that the model has good calibration, with a C statistic of 0.820 (95% CI: 0.772–0.877).

The clinical application of the nomogram prediction model for kinesophobia risk after cerebral hemorrhage involves a structured process to ensure its effectiveness and accuracy. Initially, medical staff undergo training to familiarize themselves with the model’s components and interpretation. During patient intake, relevant data including age, electronic health literacy score, NIHSS score, VAS pain score and depression score are collected through standardized assessments and medical records. Patients are then categorized into different risk levels based on the nomogram. For each patient, the predicted risk is documented, and personalized intervention plans are developed accordingly. These plans may include tailored rehabilitation programs, psychological counseling, and educational sessions to address kinesophobia. To evaluate the model’s performance, a systematic data collection framework is established. This includes tracking patient outcomes such as adherence to rehabilitation programs, improvement in motor function, and changes in kinesophobia levels over time. Additionally, feedback from medical staff regarding the model’s usability and effectiveness is gathered through surveys and interviews.

### Limitation analysis

There are some limitations in this study, including: (1) Sample limitations: The sample of this study is only from patients after brain surgery in a single third-grade A-level hospital in China. The geographical representation of the sample is limited, which may not fully reflect the true prevalence of kinesophobia in patients with cerebral hemorrhage across different regions and medical environments. To enhance the generalizability of the research findings, future studies should expand the sample source to include patients from diverse regions and hospitals of varying levels. (2) Limitations of study design: This study is a cross-sectional study, which cannot establish causal relationships between various factors and kinesophobia. Although statistical analysis reveals many associated factors, it cannot determine the direction of causality. Future research could be designed as prospective cohort studies, tracking the recovery process of patients after surgery to deeply explore the causal relationships between various factors and the development of kinesophobia. (3) Unrecognized factors: Some potential influencing factors, such as the patient’s previous surgical history and family economic status, may have been overlooked in this study. These factors may influence the occurrence of kinesophobia by affecting patients’ psychological states and access to rehabilitation resources. Future research should consider a broader range of possible factors to improve the understanding of kinesophobia in patients after cerebral hemorrhage surgery. (4) While the nomogram prediction model demonstrates promising predictive performance, its seamless integration into actual clinical workflows remains a significant limitation. The current design of the model may not be readily accessible or user-friendly for healthcare professionals, potentially hindering its widespread adoption and practical utility in clinical settings. To address this issue, future research should focus on developing convenient applications or tools that simplify the use of the model, ensuring it can be easily incorporated into daily clinical practices. This could involve creating mobile apps, electronic health record (EHR) integrations, or other digital solutions that allow medical staff to input patient data quickly and receive predictions efficiently. Additionally, providing comprehensive training and support materials for clinicians would further enhance the model’s usability and acceptance in real-world scenarios. (5) A key limitation of this study is the absence of external validation. While internal validation using the bootstrap method, it does not account for variability across different clinical settings or populations. Without testing on an independent external dataset, the model’s generalizability and real-world applicability remain uncertain and potentially overestimated. To strengthen the robustness and transportability of the model, future studies should include external validation using data from diverse and independent cohorts, ideally across multiple centers and demographic settings.

## Conclusion

In this study, the incidence of kinesophobia in postoperative patients with cerebral hemorrhage was 47.25%. Age, NIHSS score, VAS pain score and depression score were identified as independent risk factors, while the electronic health literacy score was found to be a protective factor. The nomogram prediction model based on these three indicators demonstrates good clinical applicability, discrimination, and calibration, and is highly significant for predicting the risk of kinesophobia. Therefore, clinical medical staff should pay close attention to elderly patients, dynamically assess their emotional status, NIHSS score, VAS pain score, and e-health literacy levels, enhance the dissemination of health knowledge and psychological interventions, develop personalized rehabilitation programs for patients, and encourage early rehabilitation exercises to reduce the risk of kinesophobia, promote recovery, and improve quality of life.

## Supplementary Information

Supplementary Material 1.

## Data Availability

Data utilized in this study are available upon reasonable request from the corresponding author.
