# Supplementary material for: Construction and verification of a risk prediction model for patients with kinesophobia after cerebral hemorrhage surgery
Source: BMC Neurol. 2025 Jul 12;25:289. doi: 10.1186/s12883-025-04296-0 (PMC12255136; doi:10.1186/s12883-025-04296-0)
Supplement: Supplementary file 1 — Supplementary Material 1. [file 12883_2025_4296_MOESM1_ESM.docx]

**English lanaguage version of the general information questionnaire**

1. Gender: [multiple choice questions]

| □ Male | □ Female |  |  |  |  |  |  |
| --- | --- | --- | --- | --- | --- | --- | --- |

2. Age (years old) [Fill in the blanks]

_________________________________

3. Education level [multiple choice questions]

| □ Junior high school and below |
| --- |
| □ High school, technical secondary school and above |

4. Marital status [multiple choice questions]

| □ Have a spouse |
| --- |
| □ No spouse |

5. Bleeding site [multiple choice questions]

| □ Basal nuclei |
| --- |
| □ Brainstem |
| □Ventricle of the brain |
| □ Brain lobe |
| □ Thalamencephalon |
| □ Cerebellum |

6. Type of operation [multiple choice questions]

| □ Endovascular coiling for aneurysm or craniotomy for aneurysm clipping |
| --- |
| □ Craniotomy for hematoma evacuation |
| □ Stereotactic-guided intracerebral hematoma puncture and drainage |
| □ Ventricular drainage and clot lysis |
| □ Decompressive craniectomy |

7. Residence [multiple choice questions]

| □ Cities and towns |
| --- |
| □ Village |

8. Payment method of medical expenses [multiple choice questions]

| □ Pay one's own expenses |
| --- |
| □ Medical insurance reimbursement |

9. Combined with coronary heart disease [multiple choice questions]

| □ No |
| --- |
| □ Yes |

10. Combined with respiratory diseases [multiple choice questions]

| □ No |
| --- |
| □ Yes |

11. Combined with hypertension [multiple choice questions]

| □ No |
| --- |
| □ Yes |

12. Combined with diabetes [multiple choice questions]

| □ Yes |
| --- |
| □ None |

13. Operation duration (minutes) [Fill in the blanks]

_________________________________

14. Intraoperative blood loss (ml) [fill in the blank]

_________________________________

15. Hypokalemia occurred after operation [multiple choice questions]

| □ No |
| --- |
| □ Yes |
